# In-Silico Analysis of Deleterious SNPs of *FGF4* Gene and Their Impacts on Protein Structure, Function and Bladder Cancer Prognosis

**DOI:** 10.3390/life12071018

**Published:** 2022-07-09

**Authors:** Ee Chen Lim, Shu Wen Lim, Kenneth JunKai Tan, Maran Sathiya, Wan Hee Cheng, Kok-Song Lai, Jiun-Yan Loh, Wai-Sum Yap

**Affiliations:** 1 Faculty of Applied Sciences, UCSI University, Kuala Lumpur 56000, Malaysia; 1001851850@ucsiuniversity.edu.my (E.C.L.); 1001645830@usciuniversity.edu.my (S.W.L.); 1001852121@ucsiuniversity.edu.my (K.J.T.); 2 School of Pharmacy, Monash University Malaysia, Jalan Lagoon Selatan, Bandar Sunway 47500, Malaysia; sathiya.maran@monash.edu; 3 Faculty of Health and Life Sciences, INTI International University, Persiaran Perdana BBN, Putra Nilai, Nilai 71800, Malaysia; wanhee.cheng@newinti.edu.my; 4 Health Sciences Division, Abu Dhabi Women’s College, Higher Colleges of Technology, Abu Dhabi P.O. Box 41012, United Arab Emirates; lkoksong@hct.ac.ae; 5 Centre of Research for Advanced Aquaculture (CORAA), UCSI University, Kuala Lumpur 56000, Malaysia; 6 He & Ni Academy, Office Tower B, Northpoint Mid Valley City, Kuala Lumpur 59200, Malaysia

**Keywords:** FGF4, bladder cancer, prognosis, biomarkers, in-silico analysis

## Abstract

Dysregulation of fibroblast growth factors is linked to the pathogenesis of bladder cancer. The role of FGF1 and FGF3 is evident in bladder cancer; however, the role of FGF4 is vague. Despite being reported that FGF4 interacts with FGF1 and FGF3 in MAPK pathways, its pathogenesis and mechanism of action are yet to be elucidated. Therefore, this study aimed to elucidate pathogenic nsSNPs and their role in the prognosis of bladder cancer by employing in-silico analysis. The nsSNPs of FGF4 were retrieved from the NCBI database. Different in silico tools, PROVEAN, SIFT, PolyPhen-2, SNPs&GO, and PhD-SNP, were used for predicting the pathogenicity of the nsSNPs. Twenty-seven nsSNPs were identified as “damaging”, and further stability analysis using I-Mutant 2.0 and MUPro indicated 22 nsSNPs to cause decreased stability (DDG scores < −0.5). Conservation analysis predicted that Q97K, G106V, N164S, and N167S were highly conserved and exposed. Biophysical characterisation indicated these nsSNPs were not tolerated, and protein-protein interaction analysis showed their involvement in the GFR-MAPK signalling pathway. Furthermore, Kaplan Meier bioinformatics analyses indicated that the FGF4 gene deregulation affected the overall survival rate of patients with bladder cancer, leading to prognostic significance. Thus, based on these analyses, our study suggests that the reported nsSNPs of FGF4 may serve as potential targets for diagnoses and therapeutic interventions focusing on bladder cancer.

## 1. Introduction

Bladder cancer is the top ten most common cancer in the world and is reported to cause 573,000 new cases and 213,000 deaths worldwide in 2020 [1,2]. It affects the urothelial cells that line the urinary bladder. The non-muscle invasive bladder cancer (NMIBC) is prominent in 80% of patients, and muscle-invasive bladder cancer (MIBC) was reported in others [3]. Despite aggressive therapy, up to 50% of NMIBC patients return, and up to 30% develop MIBC [2]. This necessitates a lifetime surveillance cystoscopy to determine the original grade and stage of the disease due to its high recurrence rate.

The pathogenesis of bladder cancer has been studied in different signalling pathways. Recent studies report that upregulation of FGF19-FGFR4 signalling is crucial for carcinogenesis and cancer development. FGFR4 is an attractive target for developing a novel therapeutic, focusing on the FGF19-FGFR4 pathway [4]. MAPK activation due to FGF mutations was reported in 85% of NMIBC cases, emphasising the relevance of this pathway in bladder cancer aetiology [5]. FGFR is a highly conserved receptor tyrosine kinases family that mediates cellular proliferation, differentiation, and death [6]. It has been reported that aberrant FGF signalling promotes tumour development, promoting cancer cell proliferation and survival, and tumour angiogenesis [7]. The mammalian FGF family comprises highly conserved transmembrane tyrosine kinase receptors; *FGFR1*, *FGFR2*, *FGFR3*, and *FGFR4*. The most prevalent mutation in BC has been reported for *FGFR1* and *FGF3* with 50–60% NMIBC and 10–15% MIBC, respectively [8,9]. The FGFR mutation is associated with a better prognosis for BC patients, including enhanced survival and a lower chance of recurrence and progression [10]. A recent clinical study reported a 40% response rate on BC treated with a FGFR inhibitor, underscoring the receptor’s potential value as a therapeutic target.

Unlike the other FGF family, the Fibroblast Growth Factor 4 (*FGF4*) gene does not increase the incidence of cancer, however, it has been reported to be associated with poor prognosis in multiple cancer types [11,12]. The poor prognosis of cancer has been associated with a burden of fatigue, shortness of breath, weight loss, muscle wasting, and pain [13]. This causes cancer progression, side effects of treatment, high levels of systemic inflammation, and malnutrition, leading to poor health-related quality of life (HRQoL).

**Figure 1:**
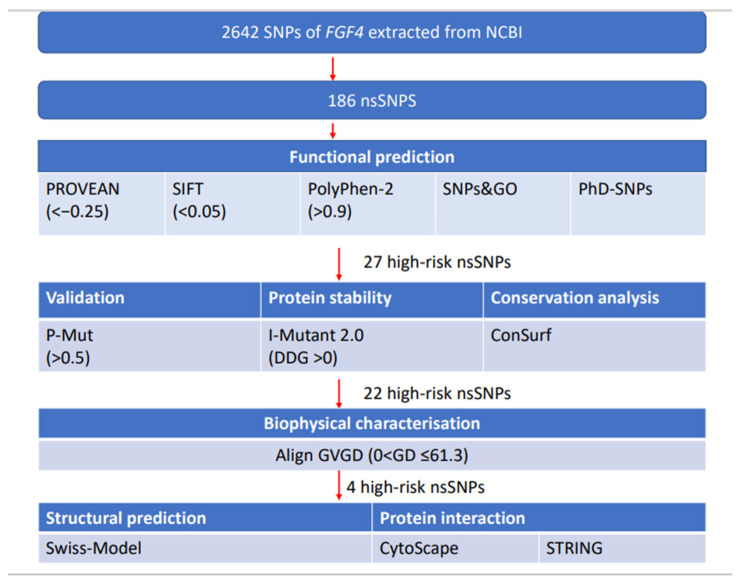
Schematic workflow for predicting high-risk deleterious nsSNPs and cancer prognosis.

The prognostic value of *FGF4* in bladder cancer remains to be elucidated, and the exact mechanism is yet to be deliberated. Here we first investigated the association between *FGF4* and poor prognosis in bladder cancer, and we have also determined the “high-risk” nsSNPs towards the pathogenesis of bladder cancer by employing an in-silico approach. The various bioinformatics steps involved in this study are shown in Figure 1.

## 2. Materials and Methods

### 2.1. Retrieving nsSNPs

The SNPs of the *FGF4* gene were retrieved from National Center for Biological Information (NCBI) dbSNP database (CRCh37.p13) and the FASTA sequence was obtained from the UniProtKB database. The data were then subjected to multiple bioinformatic tools for SNPs analysis (Figure 1).

A total of 2642 SNPs of *FGF4* (Gene ID: 2249) were retrieved from the NCBI dbSNP (https://www.ncbi.nlm.nih.gov/snp/ (accessed on 1 July 2021)), of which 186 were nsSNPs. Information on the allele change, global minor allele frequency (MAF), and residue changes were also retrieved from the database for compilation. The amino acid sequence of the *FGF4*n FASTA format was obtained from the UniProtKB (UniProt ID: P08620) (https://www.uniprot.org/uniprot/ (accessed on 1 July 2021)).

### 2.2. Identifying Deleterious nsSNPs

Deleterious nsSNPs were identified using five different in silico tools: PROVEAN (Protein Variation Effect Analyzer) [14] (http://provean.jcvi.org/index.php (accessed on 12 July 2021)) which is embedded with SIFT (Sorting Intolerant from Tolerance) [15,16] (https://sift.bii.a-star.edu.sg/ (accessed on 12 July 2021)), Polyphen-2 (Polymorphism Phenotyping v2) [17] (http://genetics.bwh.harvard.edu/pph2/ (accessed on 15 July 2021)), SNPs&GO (Single Nucleotide Polymorphism and Gene Ontology) [18] (https://snps.biofold.org/snps-and-go//snps-and-go.html (accessed on 19 July 2021)) and PhD-SNPs (Predictor of human Del Single Nucleotide Polymorphism) [18] (https://snps.biofold.org/phd-snp/PhD-SNP.html (accessed on 19 July 2021)). Only nsSNPs predicted to be damaging by all 5 tools were proceeded to further downstream analysis.

### 2.3. Validating the High-Risk nsSNPs

PMut [19] (http://mmb.irbbarcelona.org/PMut/ (accessed on 26 July 2021)) was used to screen the pathogenicity of the high-risk nsSNPs. This server utilises a predictor engine that was trained using the SwissVar 2016 entries database which contains the mutations that were considered pathogenic and also neutral which were found in over 12,000 proteins. PMut allows its user to train a custom predictor for a more precise prediction using their own datasets but for this study, the default predictor provided by the PMut was used. PMut’s predictor will give the result in the form of prediction scores that ranged from 0 to 1. Mutations with scores between 0 to 0.5 will be predicted as neutral and those scores greater than 0.5 will be predicted as pathogenic.

### 2.4. Determining Protein Stability

Protein stability was determined using I-Mutant 2.0 [20] (https://folding.biofold.org/i-mutant/i-mutant2.0.html (accessed on 3 August 2021)). The website predicts the effect of the amino acid substitution on the stability of the protein as well as the free energy value (DDG value) and the reliability index (RI) value of the amino acids. The RI value reveals the reliability of the prediction, where 0 indicates the least reliable while a 10 indicates the most reliable result. The DDG value measures the energy changes between a folded and unfolded structure. When the DDG value is higher than zero, the mutation is said to be able to increase the protein stability whereas a negative DDG value will decrease the stability of the protein.

### 2.5. Analyzing Protein Evolutionary Conservation

ConSurf [21] (https://consurf.tau.ac.il/ (accessed on 16 August 2021)) was used for determining the evolutionary conservation of each amino acid residue. It obtains the results by first running a BLAST analysis on the query protein sequence against the UNIREF-90 database and the resulting sequences were aligned using MAFFT which is a multiple sequence alignment (MSA) program that was used for creating MSA of the amino acids sequences. The MSA sequences generated were then used for constructing a phylogenetic tree and calculating the conservation rate based on an empirical Bayesian methodology. The output of this tool was given in the form of a conservation score and the prediction of the amino acid residue on whether it is exposed, buried, functional or structural. The conservation rates were then normalized and grouped into 9 different grades ranging from 1 to 9, with 1 being the most rapidly evolving position, 5 being the position of intermediate rates, and 9 being the most evolutionary conserved positions, meaning that the residue has a slower evolution rate compared to others.

### 2.6. Biophysical Characteristic Analysis with Align-GVGD

Align-GVGD (A-GVGD) [22] (http://agvgd.hci.utah.edu/ (accessed on 23 August 2021)) tool was used to predict the biological effect of missense substitutions. It predicts the transactivity by measuring the Grantham Variation (GV) which is the degree of biochemical variation among amino acids and Grantham Deviation (GD), which reflects the ‘biochemical distance’ of the mutant amino acid from the observed amino acid at a particular position [23]. The server then uses the GD value to predict whether the nsSNPS is neutral, deleterious, or unclassified. A GD = 0, is predicted as neutral, GV > 61.3 and 0 < GD ≤ 61.3, then the residue is considered as a mutant that has its composition, polarity, and volume fall close to the observed range of variation, and it will also be predicted as neutral by the server. If GV = 0 and GD > 0, this indicates the nsSNP as deleterious.

### 2.7. Analyzing Protein Interacting Network with Cytoscape

To analyze the protein interacting network, Cytoscape 3.8.2 [24] (https://cytoscape.org/ (accessed on 31 August 2021)) was employed to visualize the biological interaction between molecular complexes, modules, or pathways that possess different biological functions of the *FGF4* domain. The STRING (Search Tool for Retrieval of Interacting Genes/Proteins) interaction database [25] (https://string-db.org/ (accessed on 31 August 2021)) was used as the network source for the analysis.

### 2.8. Prediction of Structural Alteration in FGF4 Domains Using SWISS-Model

SWISS-Model [26] (https://swissmodel.expasy.org/ (accessed on 14 September 2021)) was employed to generate 3D models to predict the structural alterations in the *FGF4* domain. The nsSNPs that were identified as functional (exposed and conserved) in ConSurf and present in the FGF domain were selected for analysis.

### 2.9. Prognosis Analysis

The prognosis was evaluated using the Kaplan-Kaplan-Meier Plotter (http://www.kmplot.com// (accessed on 4 May 2022)) [27]. The survival curves and log-p values were obtained for bladder cancer.

## 3. Results

### 3.1. Predicting Deleterious nsSNPs of FGF4

Out of 186 nsSNPs, 68 nsSNPs were predicted as “deleterious” by PROVEAN, 79 “damaging” by SIFT, 88 nsSNPs were predicted as “probably damaging and possibly damaging” by PolyPhen-2, 35 nsSNPs were predicted as “disease” by PhD-SNP and 28 nsSNPs were predicted as “disease” by SNPs&GO. Analysis using PMut showed that all 27 nsSNPs scored > 0.5, suggesting pathogenicity. The results of all the six tools were integrated and 27 nsSNPs predicted as “high-risk” by all tools were proceeded with further analysis (Table 1).

### 3.2. Predicting Effects of High-Risk nsSNPs on Protein Stability

Protein stability of the nsSNPs was determined by I-Mutant 2.0 and MUPro by comparing free energy. The 27 nsSNPs predicted as “damaging” and “high-risk” were submitted to I-Mutant 2.0 and MUPro for the prediction of change in protein stability due to mutation. Twenty-three nsSNPs were predicted to have decreased protein stability by I-Mutant while MUPro determined 26 nsSNPs to have decreased stability. A total of 22 nsSNPs were predicted by both tools to decrease the protein stability of *FGF4* (Table 2).

### 3.3. Evolutionary Conservation Analysis

The evolutionary conservation of the proteins was determined using the ConSurf tool. Out of the 22high risk nsSNPs associated with decreased protein stability, four nsSNPs; rs1259280329, rs1363460000, rs1413186512, and rs930844659 were predicted to be exposed and functional. These nsSNPs possess a highly conserved sequence, indicating a slower evolutionary rate compared to other residues, therefore involved in important functional roles [28].

### 3.4. Biophysical Characteristic Analysis

Based on Align-GVGD results, the twentytwo FGF4 nsSNPs fall within class C65 (*n* = 14), class C55 (*n* = 3), class C45 (*n* = 3), class C15 (*n* = 1), and class C0 (*n* = 1). Classes of C45, C55 and C65 indicate mutations with functional impact on protein, whereas class C35 (intermediate class) along with classes of C0, C15, and C25 denote mutations with no apparent effect on protein function. The rs1259280329, rs1363460000, rs1413186512, and rs930844659 were predicted to cause functional effect on the protein.

### 3.5. Construction of Protein-Protein Interaction Network

**Figure 2:**
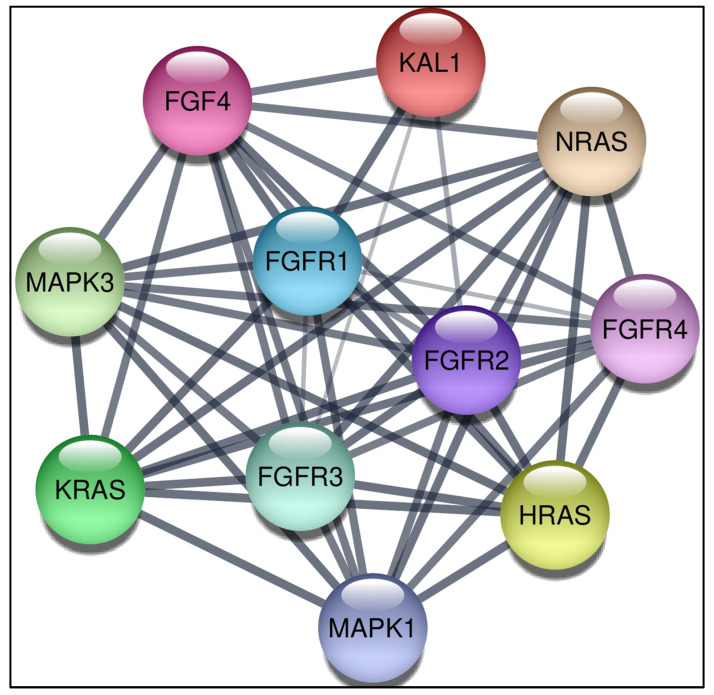
Protein–protein interaction network of FGF4 with 10 partners.

The interaction network between proteins was constructed by STRING and visualised by Cytoscape (Figure 2). The interaction consisted of 11 nodes and 49 edges. It is predicted that the FGF4 is associated with FGFR2, FGFR1, FGFR3, FGFR4, MAPK1, KRAS, MAPK3, HRAS, NRAS, and KAL1. The degree, average shortest path length, closeness centrality, betweenness centrality, and neighbourhood connectivity of these 10 proteins in interaction with FGF4 protein were also analysed using Cytoscape.

### 3.6. Prediction of Structural Alteration in FGF4 Domains Using SWISS-Model

**Figure 3:**
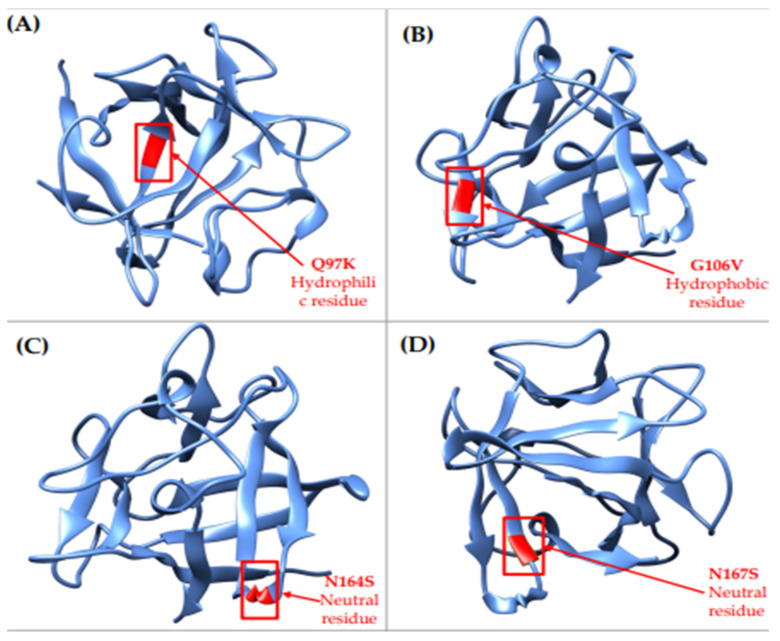
Comparison of wild-type *FGF4* protein structure with its mutant forms. (**A**) Superimposed structures of wild-type FGF4 protein with mutant protein Q97K, Glutamine into a Lysine at position 97, (**B**) Superimposed structures of wild-type FGF4 protein with mutant protein G106V, Glycine into a Valine at position 106. (**C**) Superimposed structures of wild-type FGF4 protein with mutant protein N164S, Asparagine into a Serine at position 164, and (**D**) Superimposed structures of wild-type FGF4 protein with mutant protein N167S, Asparagine into a Serine at position 167.

Mutant 3D models of rs1259280329, rs1363460000, rs1413186512, and rs930844659 localized in the FGF domain were predicted and generated using SWISS-Model (Figure 3). As hydrophobicity has a major contribution to protein function and structure, the hydrophobicity of wild-type and mutant residues were analyzed in SWISS-Model to investigate their physicochemical properties. The polarity of Q97K rs1259280329 (hydrophilic) remained unchanged throughout the mutation. Changes in polarity were observed for G106V rs1363460000 (neutral to hydrophobic), N164S rs1413186512 (hydrophilic to neutral), and N167S rs930844659 (hydrophilic to neutral).

### 3.7. Prognosis of FGF4 in Malignancies

**Figure 4:**
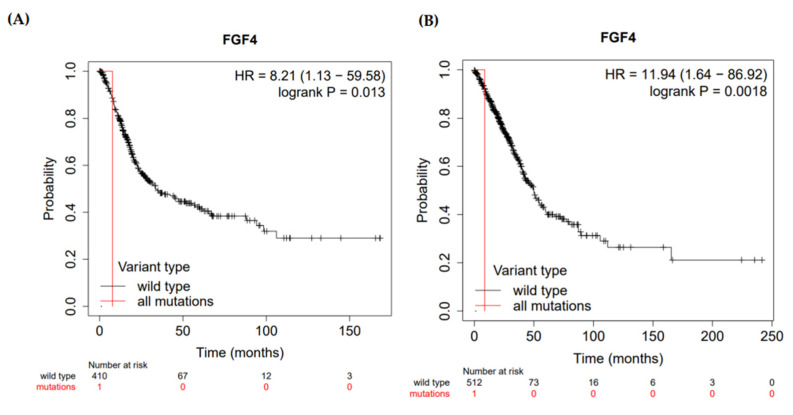
Kaplan-Meier plot showing correlation of deregulation of *FGF4* gene and overall survival rate of (**A**) bladder carcinoma and (**B**) lung adenocarcinoma patients.

A Kaplan-Meier plotter was used to determine the prognostic value of the FGF4 gene by combining gene expression and cancer patient survival. Hazard ratio (HR) with 95% confidence intervals (CI) and logrank *p*-value were calculated. *FGF4* gene showed a hazard ratio (HR) = 8.21 (95% CI, 1.13–9.58) and logrank *p*-value = 0.012 for bladder carcinoma indicating that the result was statistically significant (the relation between the high expression of the HLA-G gene and more survival rate) (Figure 4).

## 4. Discussion

Dysregulation of fibroblast growth factors, especially *FGF1* and *FGF3*, have been associated with the risk of bladder cancer [29]. However, to the best of our knowledge, the role of FGF4 in bladder cancer is limited, despite its prominent role in MAPK pathway activation linked with FGF1 and FGF3. This has created an intrigue to investigate the role of FGF4 in bladder cancer and further elucidate its role in prognosis. In this study, we have developed a pipeline (Figure 1) to determine the pathogenic nsSNPs associated with bladder cancer and its prognosis.

In this study, the nsSNPs of FGF4 were subjected to different bioinformatics tools to determine their structural and functional effect on the protein [30]. Damaging nsSNPs were predicted using five different tools, resulting in 27 nsSNPs as “highly damaging”. In order to further narrow down the number of possible pathogenic nsSNPs, P-Mut, I-Mutant, Biophysics analysis, and ConSurf tools were used to predict protein stability, the evolutionary conservation of amino acids, the physical and chemical properties, and changes in protein structure after mutations. This resulted in four “high-risk” nsSNPs; rs1259280329, rs1363460000, rs1413186512, and rs930844659, which were predicted (i) pathogenic by all five predicting tools; (ii) reduced protein stability; and (iii) evolutionary conservation showed that these nsSNPs as highly conserved. This indicates that these nsSNPs with altered protein stability may cause misfolding, degradation, or aberrant conglomeration of proteins [31]. Moreover, we also found that these highly deleterious nsSNPs with high conservation scores could increase the risk of tumorigenesis by inactivating *FGF4*.

Protein-protein interaction analysis using Cytoscape 3.8.2 indicated *FGF4* associated with *FGFR2*, *FGFR1*, *FGFR3*, *FGFR4*, *MAPK1*, *KRAS*, *MAPK3*, *HRAS*, *NRAS*, and *KAL1*. Based on the shortest average path length and the highest closeness and betweenness centrality, *FGFR1*, *FGFR2*, and *FGFR3* have the most substantial network interaction with *FGF4*. *FGF4* binds to the tyrosine kinase receptors encoded by *FGFR1* (FGFR1c), *FGFR2* (FGFR2c), *FGFR3* (FGFR3c), and *FGFR4* to begin the initiation of signalling. Loss of FGFR protein activity regulation is reported to be oncogenic leading to the overexpression of FGFR protein [32]. Another study has shown that the G388R in the protein kinase domain on *FGFR4* has been proven to increase the potential of promoting cancer cells [33].

The Q97K rs1259280329, G106V rs1363460000, N164S rs1413186512, and N167S rs930844659 predicted deleterious are located within the FGF domain, which acts as a binding site for heparan sulphate (HS) proteoglycans to trigger the activation for FGF receptors [34]. The G106V (neutral to hydrophobic), N164S (hydrophilic to neutral), N167S (hydrophilic to neutral), and G190E (neutral to hydrophobic) were predicted to change in polarity. Hydrophobic residues have a significant role in the folding of a protein chain, thus a protein needs to fold accurately to carry out its functions efficiently. Therefore, the change in polarity of G106V could potentially distort the conformation of the resulting protein. This might lead to improper binding of FGF4 to the receptors, causing incomplete signalling transduction and also the production of mutagenic proteins. According to Bellosta (2001) [35], mutated FGF4 tends to interact with *FGFR1* and produces ligands with reduced receptor binding potential. Studies have also shown that the gain in the hydrophobicity in G190E nsSNP could disrupt the MAPK pathways that are responsible for cell differentiation, as well as affect the regulations of the states of actin filaments that allow cell migration and division [31,36].

We also evaluated the *FGF4* against different types of cancer using the Kaplan-Meier bioinformatics analyses. The results indicated that the *FGF4* gene deregulation might affect the overall survival rate of patients with bladder cancer and thus affecting prognosis significance. This finding agrees with Zaharieva and colleagues (2003) who reported gene amplification of *FGF4* among bladder cancer patients.

## 5. Conclusions

In conclusion, a bioinformatics pipeline was developed to efficiently predict the deleterious effect of nsSNPs of *FGF4* on bladder cancer. We have also analysed the prognosis of *FGF4*. We believe that a similar approach can be utilised in annotating and predicting the functional and structural effect of nsSNPs on other diseases.

## Figures and Tables

**Table 1:** High risk nsSNPs predicted by the six different in silico tools.

nsSNPs ID	AA Change	PROVEAN		SIFT		Polyphen-2		PhD-SNP			SNPs&GO			PMut	
		Pred (Cut Off = −2.5)	Sc	Pred (Cut Off = 0.05)	Sc	Pred	Sc	Pred (Cut Off = 0.5)	RI	Prob	Pred (Cut Off = 0.5)	RI	Prob	Pred (Cut Off = 0.5)	Sc
rs1383383982	D75V	Del	−3.9	Dmg	0.002	Pro.dmg	0.993	Dis	1	0.610	Dis	2	0.542	Dis	0.81
rs922987433	D75Y	Del	−3.92	Dmg	0.002	Pro.dmg	1	Dis	1	0.680	Dis	4	0.538	Dis	0.81
rs760825703	R85W	Del	−5	Dmg	0.016	Pro.dmg	0.988	Dis	5	0.889	Dis	8	0.769	Dis	0.77
rs1266598072	G91D	Del	−5.56	Dmg	0.001	Pro.dmg	1	Dis	1	0.878	Dis	8	0.554	Dis	0.79
rs1194178508	G93D	Del	−5.74	Dmg	0	Pro.dmg	1	Dis	6	0.917	Dis	8	0.825	Dis	0.83
rs1250040489	G93R	Del	−6.53	Dmg	0	Pro.dmg	1	Dis	6	0.898	Dis	8	0.803	Dis	0.79
rs775542907	F94S	Del	−6.44	Dmg	0.001	Pro.dmg	0.980	Dis	7	0.888	Dis	8	0.853	Dis	0.83
rs1259280329	Q97K	Del	−3.5	Dmg	0.001	Pro.dmg	0.998	Dis	6	0.854	Dis	7	0.791	Dis	0.76
rs1469284144	I104N	Del	−6.05	Dmg	0	Pro.dmg	1	Dis	7	0.801	Dis	6	0.871	Dis	0.89
rs1363460000	G106V	Del	−8.14	Dmg	0	Pro.dmg	1	Dis	5	0.739	Dis	5	0.755	Dis	0.86
rs1432374845	L118R	Del	−4.61	Dmg	0.001	Pro.dmg	0.996	Dis	1	0.714	Dis	4	0.543	Dis	0.59
rs1245810774	G124V	Del	−8.58	Dmg	0	Pro.dmg	1	Dis	5	0.685	Dis	6	0.639	Dis	0.91
rs539419605	G124S	Del	−5.74	Dmg	0	Pro.dmg	1	Dis	3	0.793	Dis	4	0.756	Dis	0.77
rs374997743	I128F	Del	−2.91	Dmg	0.004	Pro.dmg	0.96	Dis	1	0.862	Dis	7	0.534	Dis	0.79
rs979866825	G130S	Del	−5.68	Dmg	0	Pro.dmg	1	Dis	2	0.688	Dis	4	0.578	Dis	0.74
rs966807008	S133I	Del	−5.32	Dmg	0	Pro.dmg	1	Dis	4	0.781	Dis	6	0.712	Dis	0.88
rs781699363	A138T	Del	−3.51	Dmg	0.002	Pro.dmg	1	Dis	4	0.696	Dis	5	0.52	Dis	0.87
rs757487910	M139L	Del	−2.86	Dmg	0	Pro.dmg	0.992	Dis	4	0.764	Dis	5	0.697	Dis	0.83
rs1283278927	L145P	Del	−6.29	Dmg	0	Pro.dmg	1	Dis	5	0.836	Dis	7	0.741	Dis	0.78
rs764426431	Y146C	Del	−7.41	Dmg	0.001	Pro.dmg	1	Dis	3	0.760	Dis	5	0.651	Dis	0.85
rs779058257	E154G	Del	−5.75	Dmg	0.001	Pro.dmg	1	Dis	6	0.773	Dis	5	0.785	Dis	0.82
rs756008893	C155S	Del	−9.47	Dmg	0.006	Pro.dmg	1	Dis	6	0.864	Dis	7	0.782	Dis	0.9
rs1413186512	N164S	Del	−4.73	Dmg	0	Pro.dmg	0.999	Dis	1	0.732	Dis	5	0.552	Dis	0.86
rs986306143	Y166H	Del	−4.73	Dmg	0	Pro.dmg	1	Dis	2	0.755	Dis	5	0.583	Dis	0.77
rs930844659	N167S	Del	−4.73	Dmg	0	Pro.dmg	1	Dis	2	0.562	Dis	1	0.581	Dis	0.83
rs1182350769	S171Y	Del	−5.31	Dmg	0	Pro.dmg	1	Dis	1	0.794	Dis	6	0.53	Dis	0.78
rs866953016	G190E	Del	−7.27	Dmg	0	Pro.dmg	1	Dis	4	0.680	Dis	4	0.705	Dis	0.9

**Table 2:** Overall results returned by I-Mutant 2.0, MUPro, ConSurf, and Align-GVGD.

nsSNPs ID	AA Change	I-Mutant 2.0			MUPro		ConSurf		Align-GVGD		
		Stab	RI	DDG	Stab	DDG	Pred	Sc	GV	GD	Pred
rs1383383982	D75V	Decrease	1	−1.23	Increase	0.15056579	Ex	4	0	152.01	Class C65
rs922987433	D75Y	Decrease	1	−0.99	Decrease	−0.24113434	Ex	4	0	159.94	Class C65
rs760825703	R85W	Decrease	6	−0.34	Decrease	−0.69402177	Ex	7	0	101.29	Class C65
rs1266598072	G91D	Decrease	6	−0.72	Decrease	−0.36065593	Bu	7	0	93.77	Class C65
rs1194178508	G93D	Decrease	8	−1.16	Decrease	−0.17477258	Ex	7	0	93.77	Class C65
rs1250040489	G93R	Decrease	7	−0.96	Decrease	−0.28775219	Ex	7	0	125.13	Class C65
rs775542907	F94S	Decrease	8	−2.77	Decrease	−1.5641414	Ex	4	0	154.81	Class C65
rs1259280329	Q97K	Decrease	4	−0.58	Decrease	−0.62838088	Ex & Fn	8	0	53.23	Class C45
rs1469284144	I104N	Decrease	5	−0.54	Decrease	−1.1107512	Bu	8	0	148.91	Class C65
rs1363460000	G106V	Decrease	3	−1.32	Decrease	−0.73393095	Ex & Fn	9	0	108.79	Class C65
rs1432374845	L118R	Decrease	8	−2.07	Decrease	−1.6708666	Bu	7	0	101.88	Class C65
rs1245810774	G124V	Increase	1	−0.16	Decrease	−0.49592065	Ex	7	0	108.79	Class C65
rs539419605	G124S	Decrease	7	−0.95	Decrease	−1.2395925	Ex	7	0	55.27	Class C55
rs374997743	I128F	Decrease	7	−1.88	Decrease	−1.3022244	Bu	8	0	21.28	Class C15
rs979866825	G130S	Decrease	7	−1.02	Decrease	−0.90820561	Bu	7	0	55.27	Class C55
rs966807008	S133I	Increase	1	−0.37	Decrease	−0.05605553	Bu	7	0	141.8	Class C65
rs781699363	A138T	Decrease	7	−1.15	Decrease	−1.0148323	Bu	8	0	58.02	Class C55
rs757487910	M139L	Decrease	6	−0.35	Decrease	−0.67563291	Bu & St	9	0	14.3	Class C0
rs1283278927	L145P	Decrease	8	−1.92	Decrease	−1.9056486	Bu	8	0	97.78	Class C65
rs764426431	Y146C	Decrease	1	0.15	Decrease	−0.46046358	Ex	7	0	193.72	Class C65
rs779058257	E154G	Decrease	4	−0.61	Decrease	−0.9707573	Ex	7	0	97.85	Class C65
rs756008893	C155S	Decrease	6	−1.37	Decrease	−1.4335656	Bu & St	9	0	111.67	Class C65
rs1413186512	N164S	Decrease	6	−0.69	Decrease	−0.86604139	Ex & Fn	9	0	46.24	Class C45
rs986306143	Y166H	Decrease	8	−1.68	Decrease	−1.3498789	Bu & St	9	0	83.33	Class C65
rs930844659	N167S	Decrease	5	−1.06	Decrease	−0.72801042	Ex & Fn	9	0	46.24	Class C45
rs1182350769	S171Y	Increase	4	0.96	Decrease	−1.0600288	Ex & Fn	9	0	143.11	Class C65
rs866953016	G190E	Increase	3	0.64	Decrease	−0.34251717	Ex & Fn	9	0	97.85	Class C65

**AA**: Amino acid; **Pred**: Prediction; **Sc**: Score; **RI**: Reliability Index; **Stab**: Stability prediction; **DDG**: Free energy change; **RMSD**: Root mean square deviation; **Dis**: Disease; **Ex**: Exposed; **Fn**: Functional; **Bu**: Buried; **St**: Structural; **GV**: Grantham Variation; **GD**: Grantham Deviation.

## Data Availability

All data available in this manuscript.
